# A Systematic Review of the Impact of Physicians’ Occupational Well-Being on the Quality of Patient Care

**DOI:** 10.1007/s12529-015-9473-3

**Published:** 2015-03-03

**Authors:** Renée A. Scheepers, Benjamin C. M. Boerebach, Onyebuchi A. Arah, Maas Jan Heineman, Kiki M. J. M. H. Lombarts

**Affiliations:** 1 Professional Performance Research Group, Center for Evidence-Based Education, Academic Medical Center, University of Amsterdam, Meibergdreef 9, PO Box 22700, 1100 DE Amsterdam, The Netherlands; 2 Department of Epidemiology, The Fielding School of Public Health, University of California, Los Angeles (UCLA), Los Angeles, CA USA; 3 UCLA Center for Health Policy Research, Los Angeles, CA USA; 4 Academic Medical Center, University of Amsterdam, Amsterdam, The Netherlands

**Keywords:** Occupational well-being, Job satisfaction, Physicians, Quality of patient care, Patient satisfaction

## Abstract

**Background:**

It is widely held that the occupational well-being of physicians may affect the quality of their patient care. Yet, there is still no comprehensive synthesis of the evidence on this connection.

**Purpose:**

This systematic review studied the effect of physicians’ occupational well-being on the quality of patient care.

**Methods:**

We systematically searched PubMed, Embase, and PsychINFO from inception until August 2014. Two authors independently reviewed the studies. Empirical studies that explored the association between physicians’ occupational well-being and patient care quality were considered eligible. Data were systematically extracted on study design, participants, measurements, and findings. The Medical Education Research Study Quality Instrument (MERSQI) was used to assess study quality.

**Results:**

Ultimately, 18 studies were included. Most studies employed an observational design and were of average quality. Most studies reported positive associations of occupational well-being with patient satisfaction, patient adherence to treatment, and interpersonal aspects of patient care. Studies reported conflicting findings for occupational well-being in relation to technical aspects of patient care. One study found no association between occupational well-being and patient health outcomes.

**Conclusions:**

The association between physicians’ occupational well-being and health care’s ultimate goal—improved patient health—remains understudied. Nonetheless, research up till date indicated that physicians’ occupational well-being can contribute to better patient satisfaction and interpersonal aspects of care. These insights may help in shaping the policies on physicians’ well-being and quality of care.

**Electronic supplementary material:**

The online version of this article (doi:10.1007/s12529-015-9473-3) contains supplementary material, which is available to authorized users.

## Introduction

Well-being of physicians is a growing concern [1, 2]. Compared to the general working population, many physicians suffer from burnout [3–5], as they deal with high levels of work strain and emotional demands [2]. Physicians’ well-being is vital not only to the individual physician, but also to their ability to provide high-quality patient care [2]. That is, research indicated that physicians who suffer from burnout provide less adequate patient care [6–8] and low levels of physicians’ well-being could lead to suboptimal performing health care systems [2]. Reversely, physicians with higher levels of well-being tend to provide better patient care [9]. In particular, higher levels of physicians’ satisfaction or commitment with work are associated with higher levels of patient satisfaction as well as better prevention and disease management by physicians [10, 11].

High levels of work-related well-being can be conceptualized as occupational well-being, which is defined as a positive experience with or evaluation of one’s work [12, 13], involving satisfaction, commitment, involvement, or engagement [14–16]. As such, occupational well-being distinctively involves positive indicators of work experience, instead of negative indicators, such as burnout. Naturally, occupational well-being is vital to the daily practice of physicians and physicians can be energized and satisfied in providing their patients with the most appropriate treatment [17, 18]. Indeed, many physicians experience high levels of job satisfaction and work engagement [19, 20].

Most research on physicians’ well-being has so far focused on negative indicators. In line with the positive psychology approach [21, 22], it would provide comprehensive insight when additionally understanding the impact of positive occupational well-being of physicians. Research indicated that physicians with higher levels of work satisfaction deliver better patient care, possibly because they are motivated to make every effort for their patients [9]. Occupational well-being is accompanied by more positive emotions, energy, and concentration [12, 13, 23], and it is likely that physicians who experience more well-being, energy, and concentration in their work can more easily dedicate their full attention to patients’ needs and provide them with optimal care. However, there is still no comprehensive synthesis of the evidence on the connection between physicians’ occupational well-being and patient care quality. Therefore, it remains unclear if and which aspects of patient care quality are affected by occupational well-being of physicians. We conducted a systematic review of the effects of physicians’ occupational well-being on the different aspects of quality of care.

## Method

Before starting the review, all authors agreed upon the eligibility criteria, search strategy, study selection, data extraction, and quality assessment. The review process was reported according to the Preferred Reporting Items for Systematic Reviews and Meta-Analyses (PRISMA) standards [24].

### Eligibility Criteria

Studies were considered to be eligible, when they examined the association between physicians’ occupational well-being and the quality of patient care. This resulted in the following eligibility criteria. First, the study included empirical data; non-empirical articles, such as letters, comments, and editorials, were excluded. Second, physicians had to comprise the entire sample or results had to be available for physicians as a subgroup. Third, in order to study our research question on the impact of occupational well-being on patient care quality, we included only articles that conceptualized occupational well-being as predictor or exposure variable and patient care quality as the outcome variable. Non-English language articles were not included.

### Data Sources and Searches

We searched the electronic databases MEDLINE, Embase, and PsycINFO from inception until August 12, 2014. A preliminary search was conducted with assistance of a clinical librarian to develop our search strategy and specify our keywords. We used both free text and MeSH (MEDLINE) or thesaurus (Embase and PsycINFO) terms on the following subjects: physicians, occupational well-being, and quality of patient care.

Occupational well-being was defined as a positive experience with or evaluation of work [12, 13] and was searched with the terms job satisfaction, career satisfaction, professional satisfaction, job commitment, and work engagement. To reduce the chance of missing any relevant articles, we also included several synonyms (see Additional file).

We used the definition for quality of care provided in a framework introduced by Donabedian (1966) [25] which is widely used in quality of care research [26]. The framework distinguishes three elements of patient care quality: the quality of the structures (organizational factors of the health care system), processes (actual delivery of patient care), and outcomes of patient care (consequences of delivered care) [25]. For this review, we only included processes and outcomes of care, as the structure element of the Donabedian framework focuses on the system of patient care and not on individual physicians’ delivery of patient care. Based on the definitions of processes and outcomes of patient care [25], we included the following search terms: patient centeredness, patient satisfaction, patient enablement, patient safety, and patient health outcomes (see Additional file). Finally, we performed a hand search on references of eligible articles to obtain additional eligible studies.

### Study Selection

One author (RS) performed the search, which was duplicated by a clinical librarian. Subsequently, one author (RS) screened both title and abstract. Clearly, irrelevant studies were excluded at this point when both title and abstract did not include physicians, occupational well-being, or quality of patient care. Non-empirical articles (letters, comments, and editorials) were also excluded at this stage.

After screening, titles and abstracts of the remaining studies were independently reviewed by two authors (RS and BB). If abstracts were unavailable, the full-text article was retrieved and reviewed by one author (RS), following the same procedure as for the abstracts. If full text was unavailable as well, two authors (RS and BB) independently reviewed each title. Two authors (RS and BB) independently reviewed the full texts of all remaining articles. When no consensus was reached, a third author (KL) reviewed the article (for two studies in total).

### Data Extraction and Quality Assessment

Data on study design, participants and setting, measures and measurements, and study findings were extracted by one author (RS) and duplicated by a second author (BB). When no consensus was reached, a third author (KL) assisted.

We used the Medical Education Research Study Quality Instrument (MERSQI) [27] to assess study quality on ten criteria: study design, number of institutions, response rate, type of data, internal structure, content validity, criterion validity, appropriateness and sophistication of data analyses, and outcome level. The ten MERSQI items form six domains, each with a maximum score of 3. The possible total MERSQI score can range from 5 to 18 [27]. Validity evidence of the MERSQI showed to be strong [27, 28]. Two authors (RS and BB) independently scored five studies using the MERSQI criteria, after which they agreed upon a uniform scoring procedure.

### Data Synthesis and Analysis

We intended to perform a meta-analysis to pool the findings of studies. However, meta-analyses can only yield valid results if the heterogeneity between studies is limited. In this review, the heterogeneity between studies was large, so no meta-analysis could be performed. We presented the findings of the individual studies descriptively in the text and tables. We categorized the different findings based on the different forms of occupational well-being as well as the different patient care quality categories of the Donabedian framework (see Tables 1 and 2).

**Table 1:** Number of studies on MERSQI criteria

MERSQI criteria	Number of studies
Total of included studies	18
Study design	
Single group cross-sectional	17
Single group pretest and posttest	1
Non-randomized, two groups	–
Randomized controlled experiment	–
Institutions	
Single institution	1
Two institutions	–
More than two institutions	17
Response rate	
<50 % OR not reported	3
50–74 %	8
75–100 %	7
Type of data	
Assessment by study object	18
Objective measurement	–
Internal structure (internal consistency, interrater reliability, factor analysis)	
Not reported	11
Reported	7
Content validity	
Not reported	11
Reported	7
Relations to other variables (criterion, concurrent, and predictive validity)	
Not reported	10
Reported	8
Appropriateness of data analysis	
Data analysis inappropriate for study design or type of data	2
Data analysis appropriate for study design or type of data	16
Sophistication of data analysis	
Descriptive statistics only (frequencies, measures of central tendency)	2
Beyond descriptive analysis (comparisons, correlations, relationships between variables)	16
Highest outcome level	
Satisfaction, attitudes, perceptions	12
Knowledge, skills	–
Behaviors	6
Patient/health care outcomes	–

**Table 2:** Overview on the direction of the effects of occupational well-being on aspects of patient care quality found in the eligible studies

	Processes of care					Outcomes of care
	Technical aspects	Interpersonal aspects	Patient satisfaction	Patient adherence to treatment	Overall processes	Patient health
Positive association	Job satisfaction					
	Melville et al. [29] Williams et al. [30]	Grol et al. [31] Perez-Carceles et al. [32]	Grembrowski et al. [33] Haas et al. [34] Mache et al. [35] Szenenyi et al. [36]	Dimatteo et al. [37]	Conway et al. [38] Williams et al. [30]	–
	Career satisfaction					
	Frank et al. [39]		DeVoe et al. [40]	–	Deshpande et al. [41]	–
	Work engagement					
	Prins et al. [42]	–	–	–	–	–
No association	Job satisfaction					
	Grol et al. [31] Linzer et al. [43] Utsugi-Ozaki et al. [44] Winefield et al. [45]	–	Weng et al. [46]	–		Grembrowski et al. [33]
Negative association	–	–	–	–	–	–

Studies that appear more than once in this table analyzed multiple associations between multiple variables

## Results

### Search Results

**Fig. 1:**
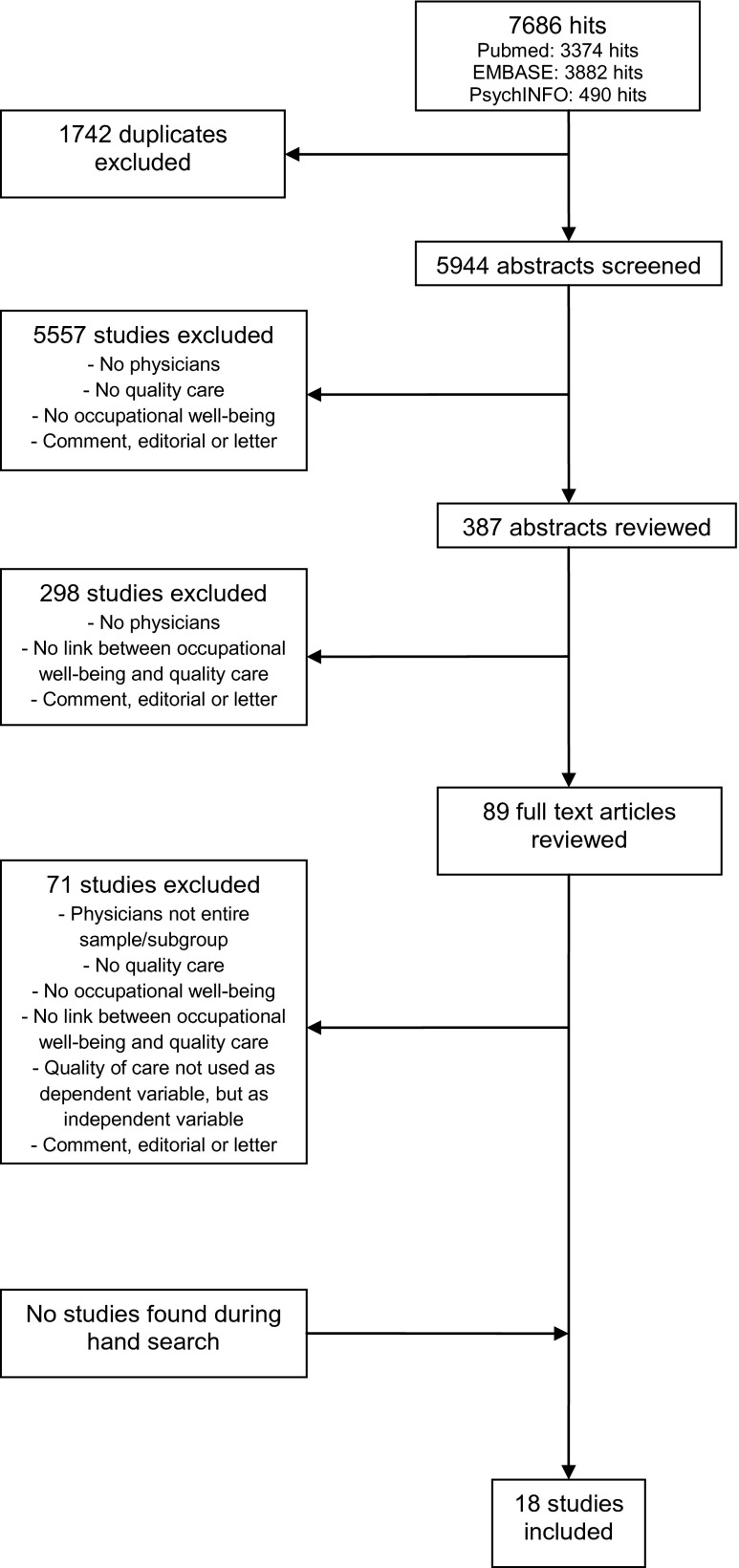
Flow chart of the review process

The search yielded 5944 unique hits (see Fig. 1, flow chart). Screening of title and abstract resulted in 387 potentially eligible articles. After abstract review, 89 articles remained and were independently reviewed and discussed on their full text. Finally, our systematic search resulted in 18 included articles. Hand search did not result in additional articles.

### Study Quality

The quality of studies ranged between 6.5 and 13 on the MERSQI scale, and the average quality was 9.8 (Table 1). Most studies had a cross-sectional design and included more than two medical centers (17 studies [29–46], see Table 1). Seven studies had a high response rate (75–100 %) [33, 34, 36, 41, 43–45], eight studies had a response rate between 50 and 75 % [29, 31, 32, 35, 37, 39, 40, 46], and three studies had a response lower than 50 % or did not report it at all [30, 38, 42]. Furthermore, seven studies reported internal structure of the measures on occupational well-being [29, 36, 38, 42, 45–47]. For patient care measures, seven studies used patient-reported data [33–37, 40, 46], seven studies used self-reported data [30, 32, 38, 39, 41, 42, 45], two studies used medical records [29, 44], and two studies used observations [31, 43]. We reported the study outcomes descriptively, with detailed quantitative results of individual studies (Table 3).

**Table 3:** Occupational well-being and quality of patient care

First author and publication year	Country	Study design	Participants and setting	Occupational well-being measurement	Quality of care measurement	Data analysis	Results	MERSQI	Content validity reported
Processes of care—technical aspects									
Work satisfaction									
Grol 1985 [31]	Netherlands	Cross-sectional survey	57 family physicians of one-man practices	Work satisfaction: positive feelings about work (24 self-reported items on, using a five-point scale)	Medical care: degree of avoidance of superfluous or insufficient medical care (observed and audiotaped consultations by four assessors)	Correlations	Work satisfaction was not associated with avoidance of superfluous (*r* = 0.19, *p* > 0.05) and insufficient medical care (*r* = 0.04, *p* > 0.05).	10.5	Yes
Linzer 2009 [43]	USA	Cross-sectional survey	422 general internists and family physicians and 1795 patients from 119 practices	Job satisfaction: overall (one self-reported item on a five-point scale)	Quality of care: control of blood pressure for hypertension, control of hemoglobin A1c and blood pressure for diabetes, stability of signs and symptoms for heart failure (audio-recorded visits) Treatment errors: missed treatment opportunities, inattention to behavioral factors, guideline non-adherence, and defined prevention errors (audio-recorded visits)	Regression analyses	There were no significant associations between job satisfaction and quality of care for hypertension (regression coefficient = −1.46, CI −8.87, 5.94) and diabetes patients (regression coefficient = 2.46, CI −3.38, 8.67), or treatment errors (regression coefficient = −1.66, CI −3.77, 0.44)	11	Yes
Melville 1980 [29]	UK	Cross-sectional survey	124 family physicians	Job satisfaction: overall (five self-reported items on a five-point scale)	Dysfunctional prescribing: prescription of practolol, monoamine oxidase inhibitors, barbiturate hypnotics, anti-infective agents acting locally on the intestinal tract, major tranquillizer in low doses, and central nervous system stimulants (databased)	*T* tests and correlations	For practolol, non-prescribers had higher job satisfaction (*M* = 12.7) than high prescribers (*M* = 10.5, *p* = 0.025). For anti-infective agents acting locally on the intestinal tract, non-prescribers had higher job satisfaction (*M* = 12.5) than high prescribers (*M* = 10.7, *p* = 0.025). The higher job satisfaction, the lower prescription of major tranquillizers: *r* = −0.327, *p* < 0.05 No significant associations for barbiturate hypnotics and central nervous system stimulants.	10.5	No
Utsugi-Ozaki 2009 [44]	Japan	Cross-sectional survey	57 general internists and 568 patients of 13 hospitals	Job satisfaction: overall (five self-reported items on a five-point scale)	Quality of care: five quality of care indicators (QI) on medical behaviors for hypertension, six QIs for type 2 diabetes, five QIs for asthma, and four QIs for cross-cutting care.	Linear regression analyses	Physician job satisfaction was not correlated with overall quality of care (regression coefficient = 0.3, CI = −3.1–3.7). Job satisfaction was not significantly related to the quality scores of hypertension (regression coefficient = −3.0, *p* = 0.22), type 2 diabetes (regression coefficient = 2.5, *p* = 0.44), asthma (regression coefficient = 8.0, *p* = 0.21), or cross-cutting care (regression coefficient = −0.4, *p* = 0.76).	11	Yes
Williams 2007 [30]	USA	Cross-sectional survey	426 physicians of 101 ambulatory clinics	Job satisfaction: overall (five self-reported items on a five-point scale)	Suboptimal patient care: error likelihood in therapeutic and diagnostic practices (nine self-reported items on a five-point scale)	Correlations	Physician job satisfaction was significantly negatively related to error likelihood (*r* = −0.12, *p* < 0.05).	8.5	Yes
Winefield 2002 [45]	Australia	Cross-sectional survey	30 family physicians	Job satisfaction: overall (one item on a seven-point scale)	Medical mistakes: mild, moderate, or severe (e.g., a preventable stroke) (one self-reported item: “describe a recent event where your patient care was or may have been affected by work stress”)	Fisher exact test	Between physicians who made mistakes that had mild, moderate, or severe actual and potential consequences, there was no difference in job satisfaction.	10	No
Career satisfaction									
Frank 2000 [39]	USA	Cross-sectional survey	4501 female physicians	*Career satisfaction* (item(s) not reported)	Prevention: counseling on mammography and counseling on clinical breast examination for 50–75-year-old patients (two self-reported items on a seven-point scale)	Chi-square tests	More satisfied female physicians were more likely to counsel regarding mammography (*p* = 0.048)	9.5	No
Work engagement									
Prins 2009 [42]	Netherlands	Cross-sectional survey	2115 residents	Work engagement: dedication, vigor, and absorption with work (15 self-reported items on a seven-point scale)	Medical errors: errors due to action/inexperience or errors due to lack of time (six self-reported items on a five-point scale)	Correlations and *T* tests	Work engagement was negatively correlated to action/inexperience errors (*r* = −0.07, *p* < 0.001) and errors due to lack of time (*r* = −0.22, *p* < 0.001). Highly engaged residents reported significantly fewer action/inexperience errors (*t* = 2.48, *p* = 0.013) and errors due to lack of time (*t* = 6.54, *p* = 0.001) than residents who were not highly engaged.	10.5	Yes
Processes of care—interpersonal aspects									
Work satisfaction									
Grol 1985 [31]	Netherlands	Cross-sectional survey	57 family physicians of one-man practices	Work satisfaction: positive feelings 10389-015-0661about work (24 self-reported items 10389-015-0661on, using a five-point scale)	Non-somatic care: explaining treatment, being open with the patient, attention to psychosocial aspects (observed and audiotaped consultations by four assessors)	Correlations	Practitioners’ work satisfaction was positively related to being open with the patient (*r* = 0.29, *p* < 0.05) and attention to psychosocial aspects (*r* = 0.28, *p* < 0.05). Practitioners’ work satisfaction was not significantly associated with explaining treatment (*r* = 0.01, *p* > 0.05).	10.5	Yes
Perez-Carceles 2006 [32]	Spain	Cross-sectional survey	227 family physicians of 52 primary care practices	Job satisfaction on the domains: daily 5practice, belonging to a professional 9group, salary and conduct of their immediate 9supervisors (four self-reported items on a 9five-point scale)	Informing patients: frequency of informing patients on diagnosis, prognosis, treatment, complementary examinations, and the work and social/family impact of the illness process (five self-reported items on a five-point scale).	Chi-square tests	Doctors who always informed their patients on diagnosis had higher scores on satisfaction with salary (*p* = 0.02), satisfaction with daily clinic (0.04) and satisfaction with belonging to a professional group (*p* = 0.04). Doctors who always informed their patients on prognosis had higher scores on satisfaction with salary (*p* =0.0001), satisfaction with daily clinic (*p* = 0.002) and satisfaction with performance of immediate superiors (*p* = 0.002). Doctors who always informed their patients on treatment had higher scores on satisfaction with belonging to a professional group (*p* = 0.001) and satisfaction with performance of immediate superiors (*p* = 0.001). Doctors who always informed their patients on complementary examination had higher scores on satisfaction with belonging to a professional group (*p* = 0.0001) and satisfaction with performance of immediate superiors (*p* = 0.009). Doctors who always informed their patients on social-professional and family impact had higher scores on satisfaction with belonging to a professional group (*p* = 0.001).	7.5	No
Processes of care—patient satisfaction									
Work satisfaction									
Grembrowski 2005 [33]	USA	Cross-sectional survey and 6-month follow-up	261 family physicians and 2004 of their patients of 72 private practices	Job satisfaction on the domains: personal autonomy, salary, volume of patients, practice management, patient care and work setting overall (six self-reported items on a five-point scale)	Patient satisfaction: overall quality of care according to pain and depression patients (one patient-reported item on a six-point scale), trust and confidence (one patient-reported item on a five-point scale) and continuity of care (one patient-reported item on a five-point scale)	Logistic regression analyses	Physician job satisfaction was not associated with patient ratings of overall quality of care (coefficients not reported). For pain patients, greater physician job satisfaction was associated with greater patient trust (coefficient = 0.06, *p* = 0.034) and greater continuity of care (odds ratio = 1.64, *p* = 0.000). For depression patients, greater physician job satisfaction was associated with higher patient ratings of overall quality of care (coefficient = 0.14, *p* = 0.041) and higher patient trust (coefficient = 0.10, *p* = 0.024).	9	No
Haas 2000 [34]	USA	Cross-sectional survey	166 general internists and 2620 patients of 11 general internal medicine practices	Professional satisfaction: overall (one self-reported item)	Patient satisfaction: overall and satisfaction with the most recent physician visit (four vs. five patient-reported items on a five-point scale)	Generalized estimation equations (multilevel linear regression analyses)	Patients of physicians with high job satisfaction had higher overall satisfaction (regression coefficient = 2.10, CI = 0.73, 3.48) and higher physician visit satisfaction (regression coefficient = 1.23, CI = 0.26, 2.21) than patients of physicians with low job satisfaction.	9	No
Mache 2012 [35]	Germany	Cross-sectional survey	98 surgeons and 122 of their patients of seven General and Visceral Surgery hospital departments	Job satisfaction: overall (one self-reported item)	Patient satisfaction (12 patient-reported items on a five-point scale)	Correlations	There was a correlation between job satisfaction and patient satisfaction (*r* = 0.49, *p* < 0.01).	9.5	Yes
Szecsenyi 2011 [36]	Germany	Cross-sectional survey	676 family physicians (practice principals), 305 physician colleagues (trainees and permanently 7employed physicians) and 47,168 of their patients of 676 primary care practices	Job satisfaction on the domains: amount of variety in job, opportunity to use abilities, freedom of working method, amount of responsibility, physical working condition, hours of work, income, recognition for work, colleagues and fellow workers, and overall job satisfaction (ten self-reported items on a seven-point scale)	Patient satisfaction (23 patient-reported items on a five-point scale) on the domains: evaluation of the physician, evaluation of the organization of the practice, intention not to change practice	Correlations	No significant correlations were found between patient satisfaction and the job satisfaction of the practice principal (*r* = 0.026, *p* = 0.497) or the physician colleagues (*r* = 0.046, *p* = 0.456). There was a significant correlation between satisfaction of the physician colleagues and patients’ satisfaction with the organization of the practice (*r* = 0.17, *p* = 0.004), while there was no significant correlation between job satisfaction of the practice principal and patients’ satisfaction with the organization of the practice (*r* = 0.018, *p* = 0.638). There were no significant correlations between job satisfaction of the practice principal or the physician colleagues and patients’ intention to leave practice (*r* = 0.045, *p* = 0.249 and *r* = 0.001 and *p* = 0.998, respectively).	12	Yes
Weng 2011 [46]	Taiwan	Cross-sectional survey	110 internists and 2872 of their patients	Job satisfaction : overall (three self-reported items on a five-point scale). Item 1: “All in all I am satisfied with my job.” Item 2: In general, I do not like working here.” Item 3: “In general, I like working here.”	Patient satisfaction: satisfaction with care provided by the doctor and the degree that a patient would recommend the doctor to friends and family members (two patient-reported items on a seven-point scale)	Correlations	Physician job satisfaction items were not significantly related to patient satisfaction with care (item 1: *r* = −0.16, item 2: *r* = −0.23, item 3: *r* = −0.16, *p* > 0.01). Physician job satisfaction items were not significantly related to patient satisfaction in terms of recommending the doctor to patients and family members (item 1: *r* = −0.06, item 2: *r* = −0.09, item 3: *r* = −0.06, *p* > 0.01).	10.5	No
Career satisfaction									
DeVoe 2007 [40]	USA	Cross-sectional survey	37.238 physicians and 179.127 patients	Career satisfaction: overall (one self-reported item on a five-point scale)	Patient satisfaction (six patient-reported items on a five-point scale)	Correlations	Physicians’ career satisfaction significantly correlated to patient satisfaction (*r* = 0.628, *p* < 0.001)	8.5	No
Processes of care—patient adherence to treatment									
Work satisfaction									
DiMatteo 1993 [37]	USA	Cross-sectional survey	186 physicians and 2546 patients of multiple HMO and solo-practices	Job satisfaction: overall (four self-reported items on a 0-100 scale)	Patient adherence to recommended medication, exercise and diet (five patient-reported items on a 0–100 scale)	Linear regression analyses	Physicians’ job satisfaction was significantly associated with patient adherence (*β* = 0.23, *p* < 0.05).	9.5	No
Processes of care—overall									
Work satisfaction									
Conway 1998 [38]	USA	Cross-sectional survey	161 physicians of an urban, public hospital	Job satisfaction (NR self-reported items on a five-point scale)	Quality of care (self-reported items on a five-point scale, content and amount of items not reported)	Structural equation model	Job satisfaction was related to the quality of care (γ = 0.55) in the structural model.	6.5	No
Williams 2007 [30]	USA	Cross-sectional survey	426 physicians of 101 ambulatory clinics	Job satisfaction: overall (five self-reported items on a five-point scale)	Suboptimal patient care: medication errors, no discussion of treatment with patients, inadequate discharge of patients and not performing a diagnostic test because of patients’ desires (five self-reported items on a five-point scale) and error likelihood (nine self-reported items on a five-point scale)	Correlations	Physician job satisfaction was significantly negatively related to suboptimal patient care (*r* = −0.17, *p* < 0.01) and error likelihood (*r* = −0.12, *p* < 0.05).	8.5	Yes
Career satisfaction									
Deshpande 2014 [41]	USA	Cross-sectional survey	4061 physicians	Career satisfaction: one self-reported item on a five-point scale.	Quality of care: ability to provide high-quality care (one self-reported item on a five-point scale).	Ordinary-least square regression analysis	Career satisfaction was positively related to quality of care (*β* = 0.14, *p* < 0.01).	11	Yes
Outcomes of care—patient health outcomes									
Work satisfaction									
Grembrowski 2005 [33]	USA	Cross-sectional survey and 6-month follow-up	261 family physicians and 2004 of their patients of 72 private practices	Job satisfaction on the domains: personal autonomy, salary, volume of patients, practice management, patient care, and work setting overall (six self-reported items on a five-point scale)	Patient health: pain and depressive symptoms (20 patient-reported items of the symptom checklist)	Logistic regression analyses	For pain and depression patients, physician job satisfaction was not associated with any of the change in health status measures (coefficients not reported).	9	No

*r* correlation coefficient, *p p*- value, *CI* confidence interval, *OR* odds ratio, *M* mean, *β* standardized regression coefficient, *b* unstandardized regression coefficient

### Study Characteristics

The eligible studies included physicians across specialties: family medicine (nine studies [29–33, 36, 37, 43, 45]), internal medicine (four studies [34, 43, 44, 46]), and surgery (one study [35]), and five studies included a broad sample of physicians across specialties [38–42] (one study sampled both primary care and internal medicine physicians). Nine studies came from the USA [30, 33, 34, 37–40, 43], six studies came from Europe (Germany [35], Spain [32], the Netherlands [42], and the UK [29]), two studies came from Asia (Japan [44] and Taiwan [46]), and one study came from Australia [45] (Table 3).

Occupational well-being was measured with the following constructs: job satisfaction (14 studies) [29–38, 43–46], career satisfaction (three studies) [39–41], and work engagement (one study) [42]. With regard to patient care, 17 studies used process measures [29–46], which focused on *technical aspects of care* (e.g., medication errors) [29–31, 43–45], *interpersonal aspects of care* (e.g., clearly explaining treatment to patients) [31, 32], *overall processes* (a combination of technical and interpersonal aspects of care) [30, 38, 41], *patient satisfaction* [33–36, 40, 46], and *patient adherence to treatment* [37] (see Table 3). One study used both processes and outcomes as measures for patient care quality [33].

### Occupational Well-Being and Quality of Patient Care

Given the diversity of included studies, we presented an overview of the direction of the study results in Table 2. The detailed results per study are presented in Table 3.

Eight studies reported on occupational well-being in relation to technical aspects of patient care. These showed contrasting results. Specifically, physicians with higher levels of occupational well-being reported less medical errors in two studies [30, 42], while these associations were not reported in two other studies on this topic [43, 45]. In addition, physicians’ job satisfaction was not associated with avoidant or superfluous medical care in consultations [31]. Another study showed that satisfied physicians prescribed less medicine which are considered indicators of incautious prescribing [29]. Two studies showed that satisfied physicians were not more likely to perform adequate clinical procedures for hypertension patients, diabetes patients [43, 44], asthma patients, or crosscutting care [44]. Physicians satisfied with their career were more likely to counsel 50–75-year-old patients regarding mammography [39], which can be considered a quality aspect of prevention as these involve a risk group for developing breast cancer (Table 3).

With regard to interpersonal aspects of patient care, family physicians who were satisfied with their work were more open to the patient and paid more attention to psychosocial aspects [31] (see Table 3). In addition, satisfied physicians informed their patients more frequently about diagnosis, prognosis, treatment, complementary examinations, and the work and social/family impact of the illness process [32].

Five studies showed positive associations between physicians’ job or career satisfaction and patient satisfaction in various specialties, i.e., family medicine, internal medicine, and surgery [33–36, 40]. Furthermore, one study on patient adherence showed that patients of satisfied physicians adhered better to recommended medication, exercise, and diet than patients of physicians who were dissatisfied with their work [37]. Another study reported no associations between physicians’ job satisfaction and patient satisfaction [46] (Table 3).

Physicians with higher levels of job satisfaction reported less suboptimal care (i.e., inadequate patient discharge, not performing a diagnostic test because of patients’ desires, medication errors, and a lack of discussion of treatment with patients) [30]. Congruently, two studies showed that satisfied physicians reported better patient care quality than physicians who were less satisfied [38, 41].

One study researched occupational well-being in relation to patient health outcomes. This study showed that job satisfaction of physicians was not associated with patients’ self-reported pain and depressive symptoms [33].

## Discussion

This systematic review indicates that occupational well-being could positively contribute to patient satisfaction [33–36, 40], patient adherence to treatment [37], interpersonal aspects of patient care [31, 32], and the quality of overall care processes [30, 38, 41]. Contrasting findings were reported by studies on physicians’ occupational well-being and technical aspects of patient care [29–31, 39, 42–45]. The association between physicians’ occupational well-being and patient health outcomes is underexplored up till date [33].

### Explanation of Findings

The findings of this review indicate that patients of physicians with high levels of occupational well-being were more satisfied with their treatment [33–36, 40] and adhered better to treatment guidelines [37]. Physicians with higher levels of occupational well-being have a positive attitude toward work and are more likely to be optimistic and helpful to others [20]. Possibly, more satisfied and engaged physicians cross over their optimism and positive attitude to patients [48, 49] [48, 49] and leave the patient more satisfied and motivated to follow up on treatment recommendations. Ultimately, better adherence to treatment recommendations indirectly contributes to better health and well-being of patients [50]. Positive effects of occupational well-being are also visible in other health care professions. Research reported that, according to their supervisors, nurses engaged with their work perform better [51]. Also, on the long term, work engagement showed to benefit work performance [52] .

As physicians with high levels of occupational well-being experience less stress and more positive emotions [12, 13, 20], they have more energy and mental resources to direct full attention to patients. This resonates with our findings that physicians who experience high levels of occupational well-being are likely to direct more attention to patients’ psychosocial aspects [31] and inform them more frequently about the process of care and on the social impact of the illness process [32]. Also, other research showed that physicians’ well-being may positively influence interpersonal aspects of patient care, as physicians with positive affect generally talk more with patients [53].

Ultimately, the health care system is targeted at achieving better health and well-being for patients [50]. A vital and engaged physician workforce is thought to be one of the conditions under which optimal patient care can take place [2]. Strikingly, research so far failed to clarify the impact of physicians’ occupational well-being on health care’s ultimate goal—improved health of patients. In particular, only one study attempted to elucidate this issue. This study showed that occupational well-being did not affect pain and depressive symptoms of patients [33]. As this is only one study, clearly, more research is needed to draw nuanced conclusions on the impact of occupational well-being on patient health outcomes. This research could consider to involve both processes and outcomes of care, as it is reasonable to assume that occupational well-being directly affects care processes, i.e., physicians’ behaviors, which ultimately contribute to patients’ health.

Although most research on occupational well-being in relation to aspects of patient care quality shows rather consistent results, findings for technical aspects of patient care were conflicting. Technical aspects of patient care refer to all medical or clinical behaviors that physicians undertake in their treatment for patients, i.e., prescribing medicine or performing a physical examination [25]. Our review indicates that physicians with high levels of occupational well-being show more adequate prescribing behavior [29]. Previous research—outside the scope of this review—showed that physicians with higher levels of well-being (in terms of positive affect) prescribed less medicine to patients [53]. Nonetheless, higher levels of occupational well-being did not prevent physicians from delivering superfluous medical care, i.e., care which is not necessary according to the most recent standards [31]. Other conflicting findings were also reported for technical aspects of patient care in terms of medical errors, as two studies showed positive associations of physicians’ occupational well-being with medical errors [30, 42] and two others did not [43, 45]. This could be due to the variation in measures, as medical errors may refer to various contents, ranging from missed diagnoses to guideline non-adherence. Future research on occupational well-being could benefit from standardized measures on technical aspects of patient care.

Some studies in this review studied specific aspects of patient care (i.e., informing patients); others reported overall patient care quality and did not specify the specific content or aspects of patient care quality [30, 38, 41]. These studies on overall quality consistently showed that physicians with higher levels of occupational well-being report better quality of patient care. More than the other studies included in this review, these studies used self-reported measures for patient care quality. Therefore, these findings should be interpreted with caution, as these findings could possibly be associated with so-called rose-colored glasses [54]. That is, a general positive attitude of physicians with higher levels of occupational well-being could account for the following positive perception of their own delivery of care [55]. Nonetheless, these findings on the positive impact of occupational well-being resonate with a previous review on negative consequences of physicians’ lack of well-being [2]. That is, as previous research reported negative consequences of physicians’ lack of well-being on the quality of care [2], it is not unreasonable to assume that the presence of physicians’ occupational well-being indeed induces positive effects on overall quality of care [56].

The majority of the included studies in this review focused on job satisfaction as a measure of occupational well-being; other forms of occupational well-being are understudied. For example, we only found one study on work engagement. Compared to job satisfaction, other forms of occupational well-being such as work engagement, have shown to induce larger effects on work performance in non-medical professions [57], therefore, more extensive research on these forms may be relevant for clinical practice.

### Limitations

Like many systematic reviews, our review could have suffered from publication bias [24]. Based on the MERSQI quality criteria, we could conclude that most studies were of average quality [27] and many studies were multicenter, showed reasonable response rates, and used validated measurements. Yet, some studies had limitations, such as the use of physicians’ self-reported data of patient care delivery [54]. In addition, the heterogeneity of measures of occupational well-being was large, hindering comparison of results and meta-analysis. On the other hand, both occupational well-being and quality of patient care are not one-dimensional constructs. Therefore, the heterogeneity provided a multifaceted view on occupational well-being in relation to the quality of patient care.

We included studies from many countries and different health systems. Because of the differences between health care systems, the working conditions of physicians and, ultimately, their occupational well-being could differ between systems [58, 59]. The aim of this study was to present an overview of the empirical literature on physicians’ occupational well-being in relation to quality of care. Additional research is needed to understand the (possible) variations in this link across health care systems.

### Implications

In the last decade, research and society increasingly focused on the prevention of burnout or other negative forms of physician well-being, in order to prevent physicians from delivering suboptimal patient care [2]. As an addition hereupon, this review yields starting points to enhance quality of patient care by mapping the effects of positive occupational well-being. Following the findings of this systematic review, patient satisfaction, patient adherence to treatment recommendations, and interpersonal aspects of patient care are most likely to benefit from increased occupational well-being of physicians. To that end, health care organizations could focus on creating optimal working conditions for physicians, possibly beneficial for their occupational well-being and, ultimately, quality of patient care. Future research could facilitate this process, by studying which specific working conditions positively contribute to occupational well-being of physicians. Although research already systematically summarized studies on the working conditions, work hours, shift length, night float, and protected sleep time [60, 61], there is little research on the effects of many other influential working conditions in medical practice (e.g., performance feedback and autonomy).

As patient care can increasingly be characterized by multidisciplinary teamwork [62, 63], future research could focus on how levels of occupational well-being among team members interact in producing better patient care. Positive feelings about work appear to cross over between colleagues in work teams [64, 65] and might boost quality of teamwork [66].

## Conclusions

Although there is substantial research on potential consequences of physicians’ well-being, the impact on patient care’s central goal—improved patient health—remains understudied. Nonetheless, research provided clarity on the association of occupational well-being with other aspects of patient care quality. This research found that physicians’ occupational well-being could positively contribute to patient satisfaction and the quality of interpersonal aspects of care. Therefore, physicians’ occupational well-being not only is vital to a healthy physician workforce, but also may contribute to better treatment and positive experiences of patients [50].

## Electronic supplementary material

Below is the link to the electronic supplementary material.

ESM 1 (DOC 38 kb)
