# Supplementary material for: A Systematic Review of the Impact of Physicians’ Occupational Well-Being on the Quality of Patient Care
Source: Int J Behav Med. 2015 Mar 3;22(6):683–98. doi: 10.1007/s12529-015-9473-3 (PMC4642595; doi:10.1007/s12529-015-9473-3)
Supplement: Supplementary file 1 — (DOC 38 kb) [file 12529_2015_9473_MOESM1_ESM.doc]

**Additional file.** Full search strategies for the databases PubMed, PsychINFO and EMBASE

**PubMed**

(engagement [tiab] OR job embeddedness [tiab] OR "Job Satisfaction"[Mesh] OR job satisfaction [tiab] OR employee satisfaction [tiab] OR work satisfaction [tiab] OR occupational satisfaction [tiab] OR vocational satisfaction [tiab] OR professional satisfaction [tiab] OR career satisfaction [tiab] OR staff satisfaction [tiab] OR employee involvement [tiab] OR job involvement [tiab] OR work involvement [tiab] OR occupational involvement [tiab] OR staff involvement [tiab] OR employee commitment [tiab] OR job commitment [tiab] OR work commitment [tiab] OR occupational commitment [tiab] OR staff commitment [tiab] OR employee well being [tiab] OR job well being [tiab] OR work well being [tiab] OR occupational well being [tiab] OR staff well being [tiab] OR employee health [tiab] OR job health [tiab] OR work health [tiab] OR occupational health [tiab] OR staff health [tiab] OR work motivation [tiab] OR job motivation [tiab] OR occupational motivation [tiab] OR vocational motivation OR employee motivation OR staff motivation [tiab] OR job attitude* [tiab] OR work attitude* [tiab] OR employee attitude* [tiab] OR staff attitude* [tiab] OR "Personnel Loyalty" [Mesh] )

**AND** (perform*[tiab] OR “Professional Competence"[Mesh] OR "Efficiency"[Mesh] OR clinical effectiveness[tiab] OR "Employee Performance Appraisal"[Mesh] OR in role behaviour* [tiab] OR in role behavior* [tiab] OR "Medical Errors"[Mesh] OR medical errors [tiab] OR diagnostic error* [tiab] OR “treatment outcome”[Mesh] OR patient outcome* [tiab] OR clinical outcome* [tiab] OR “Iatrogenic Disease”[Mesh] OR “Mortality”[Mesh] OR “Morbidity”[Mesh] OR “Length of Stay”[Mesh] OR “Reoperation” [Mesh] OR “patient readmission” [Mesh] OR "Postoperative Complications"[Mesh] OR "Intraoperative Complications"[Mesh:NoExp] OR “quality of health care” [Mesh:NoExp] OR quality of health care [tiab] OR “patient care” [Mesh:NoExp] OR "Professional Practice"[Mesh:noexp] OR "Outcome and Process Assessment (Health Care)"[Mesh] OR "Patient Compliance"[Mesh:NoExp] OR "Patient-Centered Care"[Mesh] OR "Professional-Patient Relations" [Mesh:NoExp] OR "Physician-Patient Relations"[Mesh] OR "Patient Safety"[Mesh] OR “safety/standards” [Mesh:NoExp] OR patient safety [tiab] OR "Patient Satisfaction"[Mesh] OR patient satisfaction[tiab] OR adverse event* [tiab] OR unintended event* [tiab] OR unintended consequence* [tiab] OR complaint*[tiab] OR guideline adherence [Mesh] OR guideline adherence [tiab] OR inappropriate prescribing [tiab] OR "Catheter-Related Infections"[Mesh] OR "Surgical Wound Infection"[Mesh] OR "Ventilator-Induced Lung Injury"[Mesh] OR "Pressure Ulcer"[Mesh] OR "Malpractice"[Mesh:NoExp])

**AND** ("physicians"[Mesh] OR physicians [tiab] OR physician [tiab] OR doctors [tiab] OR doctor [tiab] OR clinician* [tiab] OR GP [tiab] OR general practitioner [tiab] OR general practitioners [tiab] OR hospitalist*[tiab] OR anesthesiologist*[tiab] OR anaesthesiologist*[tiab] OR gynecologist*[tiab] OR gynaecologist*[tiab] OR surgeon*[tiab] OR pediatrician*[tiab] OR radiologist*[tiab] OR neurologist*[tiab] OR psychiatrist[tiab] OR resident [tiab] OR residents [tiab] OR trainee* [tiab] OR fellow [tiab])

**NOT** ("Letter"[Publication Type] OR "Comment"[Publication Type] OR "Editorial"[Publication Type] OR "Guideline"[Publication Type] OR "Case Reports"[Publication Type])

**PsychINFO**

job satisfaction/ OR job involvement/ OR "quality of work life"/ OR occupational health/ OR employee motivation/ OR employee attitudes/ OR "work (attitudes toward)"/ OR (engagement OR job embeddedness OR work embeddedness OR employee embeddedness OR staff embeddedness OR occupational embeddedness OR vocational embeddedness OR job satisfaction OR work satisfaction OR employee satisfaction OR occupational satisfaction OR vocational satisfaction OR professional satisfaction OR career satisfaction OR staff satisfaction OR practice satisfaction OR job involvement OR work involvement OR employee involvement OR occupational involvement OR vocational involvement OR staff involvement OR work commitment OR job commitment OR employee commitment OR professional commitment OR vocational commitment OR staff commitment OR job well being OR work well being OR occupational well being OR vocational well being OR employee well being OR staff well being OR job health OR work health OR occupational health OR vocational health OR employee health OR staff health OR motivation at work OR motivation to work OR work motivation OR job motivation OR employee motivation OR occupational motivation OR vocational motivation OR staff motivation OR job attitude* OR work attitude* OR employee attitude* OR staff attitude*).id. OR (engagement OR job embeddedness OR work embeddedness OR employee embeddedness OR staff embeddedness OR occupational embeddedness OR vocational embeddedness OR job satisfaction OR work satisfaction OR employee satisfaction OR occupational satisfaction OR professional satisfaction OR vocational satisfaction OR career satisfaction OR staff satisfaction OR practice satisfaction OR job involvement or work involvement OR employee involvement OR occupational involvement OR vocational involvement OR staff involvement OR work commitment OR job commitment OR employee commitment OR professional commitment OR vocational commitment OR staff commitment OR job well being OR work well being OR occupational well being OR vocational well being OR employee well being OR staff well being OR job health OR work health OR occupational health OR vocational health OR staff health OR employee health motivation at work OR motivation to work OR work motivation OR job motivation OR employee motivation OR occupational motivation OR vocational motivation OR staff motivation OR job attitude* OR work attitude* OR employee attitude* OR staff attitude*).ti,ab.

**AND**

exp job performance/ OR employee efficiency/ OR misdiagnosis/ OR vocational evaluation/ OR personnel evaluation/ OR exp treatment outcomes/ OR treatment effectiveness evaluation/ OR "death and dying"/ OR morbidity/ OR postsurgical complications/ OR treatment duration/ OR "quality of care"/ OR treatment compliance/ OR client satisfaction/ OR professional standards/ OR (job performance OR medical errors OR efficiency OR in role behaviour* OR in role behavior* OR misdiagnosis OR diagnostic errors OR medical errors OR treatment outcome* OR clinical outcome* OR patient outcome* OR quality of care OR mortality OR morbidity OR patient readmission OR length of stay OR reoperation OR iatrogenic disease OR patient satisfaction OR patient compliance OR patient centeredness OR physician patient relation* OR patient safety OR complaint* OR guideline adherence). id. OR (perform* OR in role behaviour* OR in role behavior* OR efficiency OR clinical effectiveness OR clinical outcome* OR patient outcome* OR quality of health care OR treatment outcome* OR medical error* OR diagnostic error* OR iatrogenic disease OR patient readmission OR length of stay OR reoperation OR postoperative complication* OR intraoperative complication * OR adverse event* OR patient safety OR patient satisfaction OR patient compliance OR patient centeredness OR physician patient relation* OR complaint* OR guideline adherence OR inappropriate prescribing).ti,ab.

**AND**

(exp physicians/) OR (physician or physicians OR doctor or doctors OR GP OR general practitioner OR general practitioners OR hospitalist* OR anesthesiologist* OR anaesthesiologist* OR gynecologist* OR gynaecologist* OR surgeon* OR pediatrician* OR radiologist* OR neurologist* OR psychiatrist OR resident or residents or residency OR trainee*OR fellow).id. OR (physician OR physicians OR doctor OR doctors OR GP OR general practitioner OR general practitioners OR hospitalist* OR anesthesiologist* OR anaesthesiologist* OR gynecologist* OR gynaecologist* OR surgeon* OR pediatrician* OR radiologist* OR neurologist* OR psychiatrist OR resident OR residents OR trainee*OR fellow).ti,ab.

**EMBASE**

(job satisfaction/ OR occupational health/ OR "quality of working life"/ ) OR (engagement OR job embeddedness OR work embeddedness OR employee embeddedness OR staff embeddedness OR occupational embeddedness OR vocational embeddedness OR job satisfaction OR work satisfaction OR employee satisfaction OR occupational satisfaction OR vocational satisfaction OR professional satisfaction OR career satisfaction OR staff satisfaction OR practice satisfaction OR job involvement OR work involvement OR employee involvement OR occupational involvement OR vocational involvement OR staff involvement OR organizational commitment OR organisational commitment OR work commitment OR job commitment OR employee commitment OR professional commitment OR vocational commitment OR staff commitment OR job well being OR work well being OR occupational well being OR vocational well being OR employee well being OR staff well being OR job health OR work health OR occupational health OR vocational health OR employee health OR staff health OR motivation at work OR motivation to work OR work motivation OR job motivation OR employee motivation OR occupational motivation OR vocational motivation OR staff motivation OR job attitude* OR work attitude* OR employee attitude* OR staff attitude*).kw. OR (engagement OR job embeddedness OR work embeddedness OR employee embeddedness OR staff embeddedness OR occupational embeddedness OR vocational embeddedness OR job satisfaction OR work satisfaction OR employee satisfaction OR occupational satisfaction OR professional satisfaction OR vocational satisfaction OR career satisfaction OR staff satisfaction OR practice satisfaction OR job involvement or work involvement OR employee involvement OR occupational involvement OR vocational involvement OR staff involvement OR organizational commitment OR organisational commitment OR work commitment OR job commitment OR employee commitment OR professional commitment OR vocational commitment OR staff commitment OR job well being OR work well being OR occupational well being OR vocational well being OR employee well being OR staff well being OR job health OR work health OR occupational health OR vocational health OR staff health OR employee health motivation at work OR motivation to work OR work motivation OR job motivation OR employee motivation OR occupational motivation OR vocational motivation OR staff motivation OR job attitude* OR work attitude* OR employee attitude* OR staff attitude*).ti,ab.

**AND**

(job performance/ OR medical error/ OR diagnostic error/ OR medication error/ OR surgical error/ OR therapeutic error/ OR treatment outcome/ OR iatrogenic disease/ OR mortality/ OR morbidity/ OR "length of stay"/ OR reoperation/ OR hospital readmission/ OR complication/ OR anesthesia complication/ OR blood transfusion reaction/ OR catheter complication/ OR infection complication/ OR infectious complication/ OR neurological complication/ OR perioperative complication/ OR peroperative complication/ OR postoperative complication/ OR preoperative complication/ OR ventilator induced lung injury/ OR wound complication/ OR health care quality/ OR clinical effectiveness/ OR practice guideline/ OR professional standard/ OR good clinical practice/ OR outcome assessment/ OR *patient care/ OR patient compliance/ OR doctor patient relation/ OR patient safety/ OR patient satisfaction/ OR inappropriate prescribing/) OR (job performance OR clinical performance OR clinical effectiveness OR in role behaviour* OR in role behavior* OR medical error* OR diagnostic error* OR patient outcome* OR clinical outcome* OR quality of health care OR patient safety OR patient satisfaction OR adverse event* OR unintended event* OR unintended consequence* OR complaint* OR guideline adherence OR inappropriate prescribing).kw. OR (perform* OR clinical effectiveness OR in role behaviour* OR in role behavior* OR medical error* OR diagnostic error* OR patient outcome* OR clinical outcome* OR quality of health care OR patient safety OR patient satisfaction OR adverse event* OR unintended event* OR unintended consequence* OR complaint* OR guideline adherence OR inappropriate prescribing).ti,ab.

**AND**

(exp physicians/) OR (physician or physicians OR doctor or doctors OR GP OR general practitioner OR general practitioners OR hospitalist* OR anesthesiologist* OR anaesthesiologist* OR gynecologist* OR gynaecologist* OR surgeon* OR pediatrician* OR radiologist* OR neurologist* OR psychiatrist OR resident or residents or residency OR trainee*OR fellow).kw. OR (physician OR physicians OR doctor OR doctors OR GP OR general practitioner OR general practitioners OR hospitalist* OR anesthesiologist* OR anaesthesiologist* OR gynecologist* OR gynaecologist* OR surgeon* OR pediatrician* OR radiologist* OR neurologist* OR psychiatrist OR resident OR residents OR trainee*OR fellow).ti,ab.

**NOT**

(conference abstract OR conference paper OR "conference review" OR editorial OR erratum OR letter OR note OR conference proceeding)
